# Lung perfusion imaging in nuclear medicine with ^99m^Tc: a comprehensive survey of radiopharmaceuticals

**DOI:** 10.1186/s41181-026-00434-2

**Published:** 2026-03-31

**Authors:** Hassan Zareian, Mehrnaz Mardazad, Mahshid Kiani, Nafise Pourshafagh, Fateme Karimi, Mehdi Shafee Ardestani

**Affiliations:** 1 Department of Medical Physics and Biomedical Engineering, Tehran University of Medical Sciences, Tehran, Iran; 2 Nuclear Medicine Technologist, Iran University of Medical Sciences, Tehran, Iran; 3 Department of Nuclear Pharmacy, Faculty of Pharmacy, Tehran University of Medical Sciences, Tehran, Iran; 4 Department of Medical Physics, Iran University of Medical Sciences, Tehran, Iran; 5 Department of Nuclear Medicine Technology, Faculty of Paramedicine, Babol University of Medical Sciences, Mazandaran, Iran; 6 Department of Radiopharmacy, Faculty of Pharmacy, Tehran University of Medical Sciences, Tehran, Iran

**Keywords:** Lung perfusion imaging, Macroaggregated albumin (MAA), Radiopharmaceuticals, Technetium-99m (⁹⁹ᵐTc), Starch microparticles, Chitosan

## Abstract

**Background:**

Technetium-99 m–labeled macroaggregated albumin ([⁹⁹ᵐTc]Tc-MAA) is the standard agent for lung perfusion imaging in pulmonary embolism (PE). Limitations related to particle consistency, preparation procedures, and blood-derived origin have prompted the development of alternative non-blood-derived tracers.

**Main body:**

A narrative review of studies up to August 2025 was performed. Candidate radiopharmaceuticals were evaluated for pulmonary localization, physicochemical properties, quality control characteristics, radiopharmacy practicality, kit-based preparation, and preclinical or clinical validation. Biodegradable microspheres, synthetic colloids, starch-based microparticles, and small-molecule complexes demonstrated promising lung uptake. Most tracers, however, lacked standardized preparation, kit compatibility, or validation in PE-relevant models. Starch-based microparticles emerged as the most translationally promising, showing practical workflow and favorable biodistribution.

**Conclusion:**

No non-blood-derived ⁹⁹ᵐTc tracer currently matches [⁹⁹ᵐTc]Tc-MAA for routine lung perfusion imaging. Future development requires standardized, pharmacopeia-aligned tracers, head-to-head comparisons, and systematic evaluation in early-phase clinical trials.

**Graphical Abstract:**

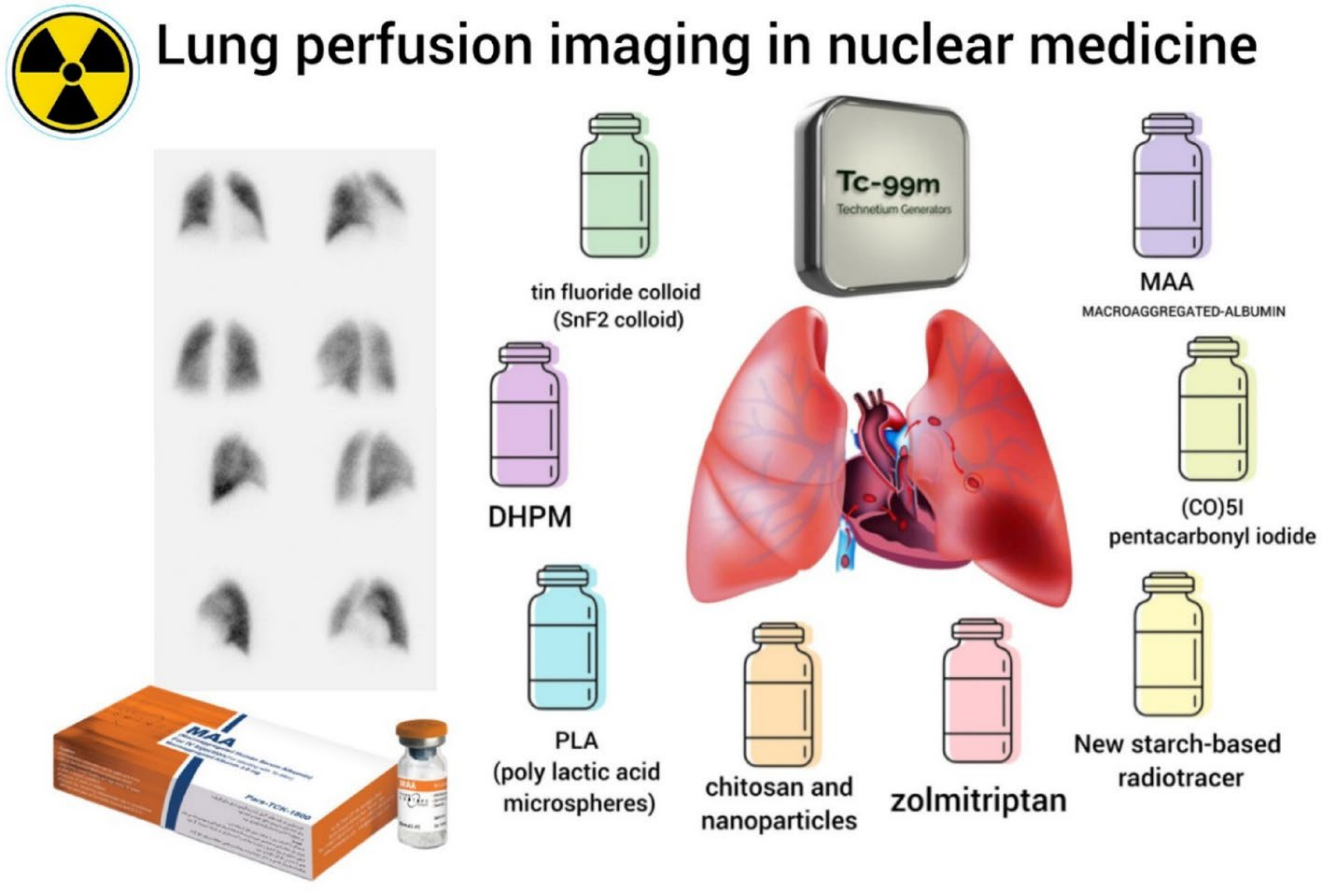

## Background

Lung perfusion imaging has long been established in nuclear medicine as a functional technique for evaluating pulmonary blood flow, regional lung function, and the physiological status of the pulmonary vasculature (Kusmirek et al. 2020, Taplin 1979). By exploiting the microvascular trapping (microembolization) of radiolabeled particles, lung perfusion scintigraphy provides quantitative and regional information, which is essential in the assessment of various pulmonary disorders, preoperative functional evaluation, and lung transplant planning (Hunt et al. 2006, Miniati et al. 1996, Delgado et al. 2000). Among these clinical applications, lung perfusion scintigraphy plays a central role in the diagnostic workup of pulmonary embolism (PE). PE is a potentially life-threatening condition, with an annual incidence of 0.03–0.2% and a three-month mortality exceeding 15% in European and U.S. populations (Mirza and Hashmi 2022, Wendelboe and Raskob 2016). Despite the increasing availability of advanced anatomical imaging modalities such as helical CT, radionuclide perfusion imaging remains a reference functional method for evaluating pulmonary circulation and detecting perfusion abnormalities associated with PE (Hunt et al. 2006).

Technetium-99 m–labeled macroaggregated albumin ([⁹⁹ᵐTc]Tc-MAA), in routine clinical use for lung perfusion scintigraphy since the early 1970s (Sajid et al. 1991), remains the most widely used radiopharmaceutical for lung perfusion imaging, typically with particle sizes in the range of ~ 10–90 μm (Bartalena et al. 2008, Zöphel et al. 2009, Vijayvergiya et al. 2009). Particles larger than 10 μm are mechanically trapped in the pulmonary capillaries during the first pass of circulation, allowing nearly 100% of the injected activity to be localized in the lungs. In contrast, particles exceeding 100 μm may obstruct larger arterioles. Particles below 30 nm may preferentially accumulate in bone marrow, whereas intermediate sizes (0.2–3 μm) tend to accumulate in the liver and spleen (Delgado et al. 2000, Malone et al. 1983). These physical properties underpin the reliability of perfusion scintigraphy for visualizing pulmonary blood flow (Delgado et al. 2000, Bartalena et al. 2008, Zöphel et al. 2009, Vijayvergiya et al. 2009).

Despite its clinical success, [⁹⁹ᵐTc]Tc-MAA is derived from human serum albumin, which has historically raised concerns regarding biological-source materials and the theoretical risk of transmitting blood-borne pathogens (e.g., hepatitis B, hepatitis C, HIV, and prion diseases) (Hunt et al. 2006, Miniati et al. 1996, Delgado et al. 2000).

However, with contemporary manufacturing controls and regulatory oversight, documented transmission events are rare, and the practical relevance of this concern in routine clinical use remains limited; in this context, Hunt et al. (Hunt et al. 2006) investigated [^99m^Tc]Tc-MAA prepared with recombinant human serum albumin (rHSA) as an alternative source material to donor-derived albumin.

In parallel, [^99m^Tc]Tc-MAA remains supported by established kit-based preparation and routine quality-control frameworks in clinical practice. At the same time, interest in alternative tracers extends beyond biological-source considerations to include standardization, workflow practicality, and formulation flexibility (Ergun et al. 2000, Perkins and Frier 1999).

Additionally, a small proportion of patients may experience adverse reactions such as wheezing, chest tightness, or nausea, which may limit use in selected populations (Hunt et al. 2006, Zöphel et al. 2009).

Taken together, these considerations, along with the need for improved standardization, reproducible specifications/QC, and translational practicality, motivate ongoing research into alternative albumin-free lung perfusion radiopharmaceuticals (Ergun et al. 2000, Perkins and Frier 1999).

Earlier studies have explored synthetic and non-human alternatives for lung perfusion imaging (Ergun et al. 2000, Perkins and Frier 1999). Poly(lactic acid) (PLA) microspheres have demonstrated promising pulmonary uptake and biodegradation profiles in preclinical studies (Ergun et al. 2000, Nijsen et al. 1999). Similarly, novel technetium-based compounds, including ⁹⁹ᵐTc-carbonyls ([⁹⁹ᵐTc]Tc(CO)₅I), ⁹⁹ᵐTc-dihydropyrimidinone (DHPM), ⁹⁹ᵐTc-tin fluoride colloid ([⁹⁹ᵐTc]Tc–SnF₂), starch-based radiopharmaceuticals ([⁹⁹ᵐTc]Tc-starch), and ⁹⁹ᵐTc-zolmitriptan have shown variable but encouraging pulmonary localization in animal models (Miroslavov et al. 2009, De et al. 2010, Tsopelas et al. 2003, Lacoeuille et al. 2010, Rashed et al. 2016). These innovations offer potential advantages such as avoidance of donor-derived materials and associated theoretical biological-source concerns, high lung retention, and prospects for ready-to-use kit formulations (Ergun et al. 2000, Perkins and Frier 1999, Miroslavov et al. 2009, De et al. 2010, Tsopelas et al. 2003, Lacoeuille et al. 2010, Rashed et al. 2016). However, despite promising preclinical results, most of these alternatives have yet to achieve widespread clinical application (Ergun et al. 2000, Perkins and Frier 1999).

The objective of this review is to summarize the current knowledge regarding lung perfusion radiopharmaceuticals, evaluate the strengths and limitations of both conventional and synthetic agents, and identify gaps in research that must be addressed to translate these compounds into clinical practice. By systematically reviewing preclinical and early clinical evidence, this work provides a comprehensive foundation for future studies aimed at developing safer, more effective, and readily applicable lung imaging agents.

### Research strategy

A narrative literature search was conducted in PubMed and Google Scholar to identify studies related to lung perfusion imaging, macroaggregated albumin (MAA), its alternatives, and relevant MeSH terms in nuclear medicine. The search included all articles published up to August 2025. We screened titles and abstracts to determine eligibility and examined the reference lists of included articles to identify additional relevant studies. The co-authors independently reviewed all potentially eligible articles. Any discrepancies in article selection were resolved through discussion and consensus. Given the limited and heterogeneous nature of the available evidence, findings from eligible studies and case series were synthesized into a structured narrative review.

Eligible candidates were appraised using four translational benchmarks: (i) mechanism/behavior in the pulmonary microcirculation, (ii) specifications and quality control (including particle-size range and administered dose units), (iii) workflow practicality, including feasibility of routine or kit-based preparation, and (iv) level of preclinical and/or clinical validation.

## Main text

We evaluated each candidate tracer using the four predefined translational benchmarks (mechanism, specifications/QC and dose unit, workflow practicality, and validation level) and summarized overall readiness in Table 1. In the following sections, each agent is discussed within this framework.

### Macroaggregated albumin (MAA)

#### Chemical aspects of [^99m^Tc]Tc-MAA

Technetium-99m-labeled macroaggregated albumin ([^99^mTc]Tc-MAA) is the established radiopharmaceutical for lung perfusion imaging. It remains the most frequently used human serum albumin (HSA)-based formulation for radiolabeling in nuclear medicine (Delgado et al. 2000, Worsley and Alavi 2003, Parker et al. 2012). The radiolabeling process involves reduction of [^99m^Tc]TcO_4_^−^ by stannous ions (SnCl_2_) in the commercial kit, enabling binding of reduced technetium to albumin aggregates (Dewanjee 1990). Proposed mechanisms include the formation of reduced technetium-oxo species coordinated to albumin aggregates (Marenco et al. 2021, Canziani et al. 2022). The resulting [^99m^Tc]Tc-MAA complex retains more than 90% of its radioactivity when incubated in human blood for up to 24 h at body temperature (37 °C) (Hunt et al. 2006).

#### Preparation of [^*99 m*^Tc]Tc-MAA

[^99m^Tc]Tc-MAA is manually produced by adding a ^99m^Tc solution to a commercially available MAA kit. The ^99m^Tc source is sodium pertechnetate ([^99m^Tc]TcO_4_^−^, Na^+^), eluted from a ^99^Mo/^99m^Tc generator.

The radiolabeling process is carried out at a pH of 6 and can be completed within approximately 15 min. This process facilitates the preparation of [^99m^Tc]Tc-MAA under Good Manufacturing Practice (GMP) conditions without requiring a heating step (Canziani et al. 2022). Following preparation, the suspension is subjected to quality control testing based on the clinical standards provided by the kit manufacturer before administration to patients. Instant thin-layer chromatography (iTLC) is typically employed to ensure the radiochemical purity. Additionally, the distribution of radioactivity among the particles is assessed by filtering the [^99m^Tc]Tc-MAA suspension through a 3 μm membrane filter and measuring the radioactivity on the filter versus that in the filtrate (Dewanjee 1990).

Before dispensing, it is essential to gently resuspend the vial, verify the radiochemical purity, and ensure the pH is within acceptable limits. To minimize imaging artifacts, precautions must be taken during injection. Standard handling precautions (gentle resuspension, avoidance of blood backflow, and slow intravenous injection under routine patient positioning/breathing conditions) are recommended to minimize aggregation-related artifacts and ensure consistent particle delivery (Mirza and Hashmi 2022, Blanc-Béguin et al. 2022).

#### Pharmacological aspects of [^*99 m*^Tc]Tc-MAA

After intravenous injection, the majority of [^99m^Tc]Tc-MAA particles are trapped in the terminal pulmonary arterioles during the first pass through the lungs. For optimal imaging, the size of these particles must be carefully controlled to match the physiological dimensions of alveolar capillaries, which average approximately 5.5 μm in diameter. Particles smaller than 10 μm may pass through the pulmonary vasculature and be phagocytosed by the reticuloendothelial system, whereas particles larger than 150 μm can obstruct larger arterioles and cause significant perfusion disturbances (Schembri et al. 2015, United States Pharmacopei , Jensen et al. 2022). In the suspension of [^99m^Tc]Tc-MAA, particle sizes typically range from 10 to 90 μm, with an average size between 20 and 40 μm. To achieve a uniform distribution of radiotracer activity that reflects regional pulmonary perfusion, suppliers recommend administering 60,000–700,000 MAA particles per dose. Particle size distribution and particle number per dose are key clinical benchmarks that any alternative perfusion agent should match to ensure reproducible trapping and acceptable safety. This standardization is particularly important given the large number of pulmonary capillaries and pre-capillary vessels that collectively determine regional trapping behavior (Suga et al. 2008).

According to the European Pharmacopeia, at least 80% of the administered radioactivity should localize in the lungs within 15 min of injection, while no more than 5% accumulates in the liver and spleen (European ). Animal studies in rabbits have confirmed that over 90% of the injected radiotracer accumulates in the lungs within minutes. Moreover, more than 80% of the radiotracer remains in the lungs during the first hour following injection (Hunt et al. 2006).

**Fig. 1:**
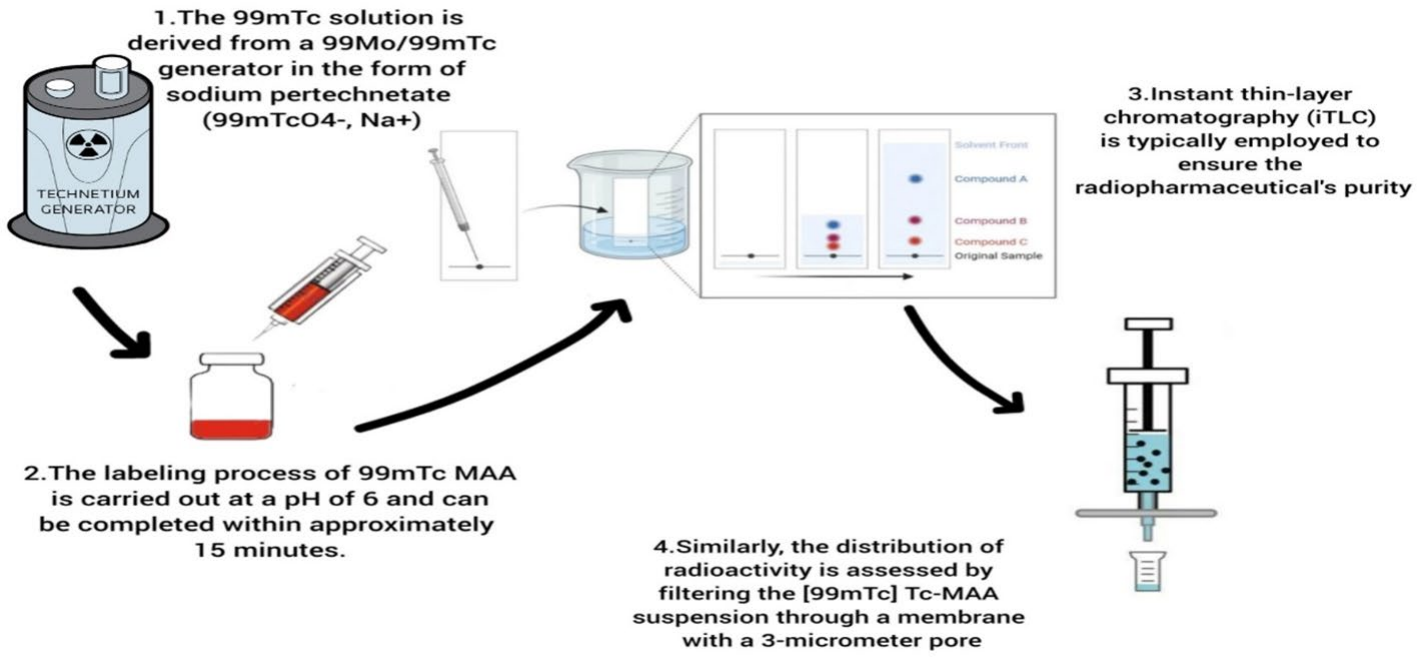
Schematic representation of the radiolabeling process for [^99m^Tc]Tc-MAA, outlining key preparation steps, reaction conditions, and quality control measures

According to a study by (Malone et al. 1983), approximately 98% of [^99m^Tc]Tc-MAA activity localized in the lungs immediately after intravenous injection. The subsequent clearance of the radiotracer from the lungs occurred in a biphasic pattern, with distinct half-lives in each phase. In the initial phase, 56% of the radiotracer exhibited a half-life of 0.88 ± 0.16 h, while the remaining 44% cleared with a half-life of 4.56 ± 0.39 h. At 3 h post-injection, uptake in the kidneys and bladder was measured at 3.6 ± 2.1% and 5.1 ± 4.0%, respectively (Malone et al. 1983). The effective half-life of [^99m^Tc]Tc-MAA is estimated to range between 3.9 and 5 h (Xiong et al. 2022). Collectively, these kinetics support a practical imaging window characterized by high initial lung trapping followed by gradual clearance, consistent with routine perfusion scintigraphy protocols. Figure 1 illustrates the radiolabeling process and preparation steps of [^99m^Tc]Tc-MAA.

Despite its clinical efficacy, [^99m^Tc]Tc-MAA presents potential safety concerns due to its radioactive nature and the intravenous administration of colloidal particles. These risks may be exacerbated in patients with pre-existing medical conditions. For instance, in individuals with severe pulmonary hypertension or a right-to-left cardiac shunt, it is essential to minimize the number of administered particles to reduce the risk of adverse effects (Zöphel et al. 2009). A significant drawback of [^99m^Tc]Tc-MAA is its reliance on human-derived albumin, which may raise regulatory, supply-chain, and acceptability considerations and motivate interest in non-biological alternatives (Whinnery and Young 1980). Therefore, the development of a non-blood-based alternative that maintains diagnostic efficacy while addressing these safety concerns is essential (Perkins and Frier 1999).

[^99m^Tc]Tc-MAA succeeded clinically because it combines high first-pass lung trapping, routine kit-based preparation with straightforward QC, and extensive human validation. Against this benchmark, the following discussion critically reviews key research efforts to identify alternative radiotracers that could address remaining limitations—particularly reliance on a human-derived component and particle-related standardization/safety considerations. Table 1 provides an overview of the most promising candidate radiotracers evaluated to date.

### Poly(lactic acid) microspheres (PLA)

In 2000, E. L. Ergün and colleagues conducted a study to evaluate the pulmonary uptake and biodegradability of poly(lactic acid) (PLA) microspheres for potential use in both radioembolization therapy and diagnostic nuclear imaging (Ergun et al. 2000). Unlike macroaggregated albumin (MAA), which is derived from blood products, PLA is a fully biodegradable synthetic polymer, offering a safer and non-blood-based alternative (Perkins 1998). This study highlights the potential of synthetic polymers and polypeptides, specifically PLA microspheres, as viable candidates for radiopharmaceutical development (Stolnik et al. 1995). The PLA microspheres were prepared using a solvent evaporation method: 6% PLA was dissolved in 5 mL of methylene chloride and emulsified in a 100 mL aqueous phase. After stirring for 2 h, the resulting microspheres were washed, filtered, and dried. The final particles ranged in size from 1.0 to 100 μm, with an average size of 39.5 μm. These findings support further evaluation of PLA-based microspheres in pulmonary imaging applications (Ergun et al. 2000).

#### Chemical aspects of [^99m^Tc]Tc‑PLA

In the aforementioned study, 20 mg of PLA microspheres were mixed with distilled water containing 0.1% Tween 80. Then, 0.2 mL of stannous chloride (SnCl_2_) and 20 mCi of sodium pertechnetate ([^99m^Tc]TcO_4_^−^) in 1 mL were added to the vial. The pH was initially adjusted to 3 using 1 N hydrochloric acid (HCl), and the mixture was rotated for 120 min to facilitate radiolabeling. Following incubation, the pH was adjusted to 7 by the addition of 0.5 N sodium hydroxide (NaOH) solution (Ercan 1992). This multi-step, low-pH, 120-minute labeling workflow may be less compatible with routine kit-based clinical preparation.

#### In vitro evaluation

Radiolabeling efficiency of [^99m^Tc]Tc-PLA microspheres was assessed using instant thin-layer chromatography (iTLC). The results showed that unbound [^99m^Tc]TcO_4_^−^ remained consistently below 2%, demonstrating a labeling efficiency greater than 98%. The radiolabeled particles also indicated excellent in vitro stability, retaining approximately 96% of radioactivity at room temperature after 24 h of storage (Ergun et al. 2000).

#### In vivo biodistribution

To assess the in vivo biodistribution, fifteen mice were intravenously injected with [^99m^Tc]Tc-PLA microspheres in 0.1 mL of suspension (the original report states “20 mCi”; this appears inconsistent with the rabbit dose and is therefore interpreted here as 20 µCi). At specific time intervals, the animals were sacrificed, and organs were removed, weighed, and analyzed for radioactivity uptake. In addition, five adult New Zealand rabbits received intravenous injections of 1 mCi of [^99m^Tc]Tc-PLA microspheres. The biodistribution pattern is reported in Table 1 of the original report (Ergun et al. 2000). In mice, lung uptake was reported as prominent relative to other organs during early time points. In rabbits, scintigraphy primarily visualized the lungs up to 24 h, supporting strong pulmonary retention (Ergun et al. 2000).

While the extended biodegradation rate of PLA microspheres may pose a limitation for diagnostic imaging in patients with lung disorders, modifications such as using lower molecular weight PLA or blending PLA with varying molecular weights may help to mitigate these issues. Slow biodegradation has been leveraged in therapeutic microsphere concepts with longer-lived β-emitters (Nijsen et al. 1999, Visscher et al. 1985, Mumper et al. 1992, Mumper et al. 1991); however, such applications are outside the scope of diagnostic ^99m^Tc lung perfusion imaging. Overall, despite certain limitations for diagnostic applications, PLA microspheres represent a versatile platform with potential utility in both diagnostic and therapeutic nuclear medicine.

#### Critical appraisal and translational relevance

Strength: Non-biological, biodegradable carrier with high reported radiolabeling efficiency and 24-h in vitro stability. Pulmonary microvascular trapping is plausible based on particle size and lung-dominant early biodistribution.

Standardization gap: Reported particle size range is broad (1–100 μm) and particle number per dose is not standardized/reported (NR), limiting safety assessment and reproducibility relative to MAA benchmarks.

Workflow barrier: Labeling requires low pH and prolonged rotation (~ 120 min) with subsequent pH adjustment, which is unlikely to be compatible with routine kit-based emergency perfusion workflows.

Evidence gap/value proposition: Evidence remains limited to a single preclinical report with no human perfusion studies; a clear clinical advantage over [^99m^Tc]Tc-MAA is not demonstrated (Ergun et al. 2000).

Overall, [^99m^Tc]Tc-PLA remains a preclinical proof-of-concept with low translational readiness for routine diagnostic lung perfusion imaging at present.

### ^99m^Tc–tin fluoride (SnF₂)

In 2006, Chris Tsopelas and colleagues conducted a study on ^99m^Tc–tin fluoride colloid (^99m^Tc–SnF_2_) to assess its potential for lung imaging (Tsopelas et al. 2006). While this radiotracer was originally developed and used clinically as a radiopharmaceutical for radiolabeling leukocytes in patients with inflammatory bowel disease in Australia, its small colloidal particle size (≈ 1–3 μm) limits its utility for pulmonary imaging due to minimal lung accumulation (Tsopelas et al. 2003). The study aimed to modify and optimize the particle size of ^99m^Tc–SnF_2_ to enhance its retention in the pulmonary vasculature, thereby improving its suitability for diagnosing lung-related conditions and expanding the applications of ^99m^Tc–tin fluoride colloid beyond gastrointestinal inflammation, particularly in nuclear medicine imaging of the lungs (Tsopelas et al. 2006).

#### Chemical aspects of [^99m^Tc]Tc-SnF_2_

In this study, the radiolabeling of technetium-99m tin fluoride colloid was conducted using the LWC Kit ½A + B (RAH Radiopharmacy, Adelaide, Australia). Vial B contained 0.64 mg of SnF_2_ in 1 mL of water for injection. To this, different activities of [^99m^Tc]Tc-pertechnetate (ranging from 20 to 1000 MBq in 0.5 to 4.0 mL of 0.9% saline) were added under sterile conditions via a breather needle. The mixture was rotated using an RSM6 suspension mixer at 40 rpm at room temperature (~ 23 °C) for 30 to 180 min (Palecanda and Kobzik 2001). To optimize the labeling efficiency and colloidal characteristics, several experimental conditions were tested, including vial atmosphere (nitrogen vs. ambient air), mixing parameters, reaction temperature (23 °C vs. 37 °C), and the addition of calcium chloride or sodium bicarbonate as stabilizing agents. The resulting radiocolloid demonstrated high radiochemical purity, with an average of 99.4 ± 0.4% (*n* = 3). Moreover, the initially clear dispersion gradually became opaque over three hours, confirming successful radiocolloid formation suitable for diagnostic imaging applications (Tsopelas et al. 2006).

#### In vitro evaluation

The radiochemical purity (RCP) of [^99m^Tc]Tc–tin fluoride colloid was assessed using thin-layer chromatography (TLC), in which free [^99m^Tc]Tc-pertechnetate migrated to the solvent front (Rf = 1.0). RCP was calculated as 100% minus the percentage of unbound pertechnetate activity measured via a gamma counter. The radioactive particle size distribution (%RPSD) was evaluated by filtering a 0.2 mL sample of the radiocolloid through polycarbonate filters with defined pore sizes (8 μm, 5 μm, and 3 μm), pre-equilibrated under appropriate conditions. Retained and eluted radioactivity were measured to determine the proportion of particles above and below the filter size. The study explored the impact of various formulation parameters, such as atmospheric conditions, mixing duration, incubation temperature, and the addition of ionic salts or oxidizing agents, on particle size distribution. The results showed that longer mixing times and using ambient air instead of nitrogen significantly increased the proportion of particles larger than 8 μm, thereby enhancing the radiocolloid’s suitability for imaging applications (Tsopelas et al. 2006).

#### In vivo evaluation

Following approved ethical guidelines, the biodistribution of [^99m^Tc]Tc–tin fluoride colloid was assessed in female Sprague-Dawley rats (130–170 g). Each animal received an intravenous injection of 1–3 MBq of radiocolloid in 0.2 mL volume. Twenty minutes post-injection, the rats were euthanized using halothane. Critical organs were excised and analyzed for radioactivity via a large-volume gamma counter. The percentage of injected dose per organ (%ID/organ) was calculated, and the data are summarized in Table 1 of the original report. Results showed that over 70% of the administered activity localized in the lungs, with optimal uptake for preparations mixed for 50 min. Both continuous mixing for 50 min and a hybrid protocol involving 30 min of mixing followed by 150 min of static incubation achieved lung uptake exceeding 87%. Hepatic accumulation ranged from 6 to 7%, while splenic uptake remained minimal (0.3–0.7%). Post-mortem gamma camera imaging confirmed significant lung localization, with distribution patterns closely resembling those of [^99m^Tc]Tc–MAA, thereby supporting the potential of [^99m^Tc]Tc–tin fluoride colloid as an effective agent for pulmonary perfusion imaging (Tsopelas et al. 2006).

A novel non-biological [^99m^Tc]Tc–colloid formulation with relatively large particle sizes has been developed, demonstrating approximately 90% pulmonary retention in rats. Compared to [^99m^Tc]Tc–MAA, low liver and spleen uptake suggests reduced interference with lower lung visualization, although further studies are needed to validate these findings. While the current preparation time of 50 min may restrict rapid use, higher concentrations of [^99m^Tc]Tc-pertechnetate could enable multiple lung perfusion doses daily. This synthetic radiopharmaceutical was prepared by a simple modification of a leukocyte labeling kit using pharmaceutical-grade components. It features 81% of particles > 5 μm, demonstrates strong potential for lung perfusion imaging, and warrants further clinical evaluation.

#### Critical appraisal and translational relevance

Strength: Synthetic, non-biological kit-based preparation with very high radiochemical purity and controllable RPSD, achieving lung trapping comparable to [^99m^Tc]Tc-MAA in a rat model.

Standardization gap: Particle number per administered dose and batch-to-batch reproducibility (RPSD consistency across preparations) are not yet established as clinical specifications.

Evidence gap: Evidence is limited to a single preclinical study (rats; post-mortem imaging) with no human lung perfusion data.

Operational consideration: Preparation requires ~ 50 min mixing, which may constrain emergency workflows unless operationally streamlined (Tsopelas et al. 2006).

Overall, [^99m^Tc]Tc–SnF₂ shows moderate translational readiness among non-biological candidates, but requires standardization (including dose particle specifications) and clinical validation.

### [^99m^Tc]Tc(CO)_5_I

In 2009, Alexander E. Miroslavov and colleagues conducted a study to evaluate the potential of [^99m^Tc]Tc(CO)₅I as a lung perfusion imaging agent, investigating its biodistribution and pharmacokinetic profile in order to address safety concerns associated with human blood-derived radiopharmaceuticals such as [^99m^Tc]Tc-MAA (Miroslavov et al. 2009). The lipophilic nature of the [^99m^Tc]Tc(CO)_5_I complex has prompted extensive investigation into its biological behavior as a potential radiopharmaceutical (Alberto et al. 2004).

Prior studies have mainly focused on the [^99m^Tc]Tc(CO)_3_(H_2_O)_3_^+^ species (Alberto et al. 2001), which serves as a common intermediate in the synthesis of technetium carbonyl complexes. Technetium pentacarbonyl halides, represented by the general formula [^99m^Tc]Tc(CO)_5_X (where X = Cl, Br, or I), are known precursors for a variety of technetium carbonyl compounds. The research group previously reported an efficient one-step synthesis method for [^99m^Tc]Tc(CO)_5_X complexes via direct carbonylation of [^99m^Tc]TcO_4_^−^ under elevated pressure and temperature conditions (Miroslavov et al. 2008).

#### Chemical aspects of [^99m^Tc]Tc(CO)_5_I

Sodium pertechnetate ([^99m^Tc]TcO_4_^−^) was combined with the appropriate quantity of hydrogen iodide (HI), followed by carbonylation under high-pressure conditions. The reaction was conducted at a pressure of 130–170 atm of carbon monoxide (CO) and a temperature range of 160–180 °C for 40–60 min. The product was isolated by releasing autoclave pressure into saline, yielding up to ~ 50% of the initial activity (Miroslavov et al. 2009). For HPLC reference purposes, non-radioactive Tc(CO)₅I was prepared separately as a crystalline standard (reported yield 60–65%), and its crystal structure has been described previously (Grigor’ev et al. 1997).

#### In vitro evaluation

The radiochemical purity of the [^99m^Tc]Tc(CO)_5_I preparation, determined by high-performance liquid chromatography (HPLC), was greater than 98%. The compound demonstrated considerable stability under acidic conditions (pH 1) and phosphate-buffered saline (PBS, pH 7.4) for at least 8 h. Over longer incubation (up to 24 h) in PBS (pH 7.4), minor conversion was observed ( ≤ ~ 10%), consistent with formation of a tricarbonyl-type species (e.g., [^99m^Tc]Tc(CO)₃(H₂O)₃⁺). However, in alkaline solutions (pH 10–12), partial oxidation of the complex was observed, resulting in the formation of free [^99m^Tc]TcO_4_^−^. Notably, no evidence of [^99m^Tc]TcO_2_ formation was detected across the studied pH range. In addition, plasma incubation (37 °C, 1 h), as assessed by iTLC, suggested that the complex remained intact, with most activity migrating from the plasma spot (≈ 78%), while residual activity at the origin (≈ 22%) was attributed to agglutination with the dried plasma matrix rather than formation of decomposition products (Miroslavov et al. 2009).

#### In vivo evaluation

To assess the biodistribution of [^99m^Tc]Tc(CO)_5_I, a dose of 0.4 MBq was administered intravenously via the tail vein to halothane-anesthetized Sprague–Dawley rats (*n* = 4). The animals were euthanized using an overdose of halothane at 15 min and 1 h post-injection, followed by cardiac puncture for blood collection. Major organs such as the lung, liver, spleen, heart, kidneys, and blood were removed, placed into labeled tubes, and weighed for radioactivity measurement (Miroslavov et al. 2009).

In parallel, three chinchilla rabbits received 10 MBq of [^99m^Tc]Tc(CO)_5_I via the auricular vein. Whole-body scintigraphy was performed using a dual-head Siemens E-CAM gamma camera. The radiotracer demonstrated rapid pulmonary uptake, with homogeneous distribution observed throughout the lung parenchyma within 30 s post-injection. Pulmonary retention of the complex remained at least 20 min (Miroslavov et al. 2009).

Dynamic imaging showed that ~ 70% of administered activity appeared promptly in the lungs and remained approximately constant over ~ 20 min; the lung fraction was ~ 60% at the end of the first pass and rose to ~ 72% by ~ 4 min before plateauing. In the original study, Table 1 presents the quantitative biodistribution data in Sprague–Dawley rats, providing a comprehensive overview of the radiotracer’s systemic behavior. At 1 h post-injection, rat biodistribution showed substantial lung activity together with measurable liver uptake, indicating that extrapulmonary distribution was not negligible (Miroslavov et al. 2009).

Although the pulmonary retention rate of [^99m^Tc]Tc(CO)_5_I does not meet the threshold set by European guidelines for [^99m^Tc]Tc-MAA, SPECT imaging indicated a uniform radiotracer distribution in the lung tissue of a healthy rabbit. The high radiochemical purity, lipophilic and neutral character, and rapid in vivo uptake profile of [^99m^Tc]Tc(CO)₅I underscore its potential as a novel lung imaging radiopharmaceutical. However, further research is necessary to enhance pulmonary retention and to optimize ^99m^Tc-pentacarbonyl complexes for clinical application in nuclear medicine.

#### Critical appraisal and translational relevance

Strength: Non-blood-derived small-molecule complex with high radiochemical purity and rapid, homogeneous lung visualization on rabbit imaging, including uniform SPECT distribution at ~ 25 min.

Mechanistic/validation gap: Lung localization is not particle-based; the mechanism is not fully established, and performance has not been demonstrated in disease models (e.g., pulmonary embolism) or in humans.

Benchmark gap vs. MAA: Reported lung extraction/retention (~ 60–70%) remains below typical pharmacopeial expectations for [^99m^Tc]Tc-MAA (≥ 80% in lungs with minimal liver/spleen), and liver activity is non-negligible in rat organ-level data.

Operational barrier: Preparation requires high-pressure/high-temperature carbonylation (autoclave), which is unlikely to be compatible with routine kit-based clinical radiopharmacy workflows (Miroslavov et al. 2009).

Overall, [^99m^Tc]Tc(CO)₅I demonstrates interesting lung affinity but currently shows low translational readiness for routine lung perfusion imaging due to manufacturing impracticality and lung retention below MAA benchmarks.

### [^99m^Tc]Tc–dihydropyrimidinone (DHPM)

In 2010, Kakali De and colleagues investigated the synthesis, technetium-99m (^99m^Tc) radiolabeling, and biological evaluation of 5-ethoxycarbonyl-4-phenyl-6-methyl-3,4-dihydro-(1 H)-pyrimidine-2-one (DHPM) as a potential radiotracer for lung perfusion imaging. This study responds to the growing interest in dihydropyrimidinones and their analogs due to their broad spectrum of pharmacological activities, including antiviral, antitumor, antibacterial, antihypertensive, and anti-inflammatory effects (Kalita and Phukan 2007). The DHPM compound was synthesized through a one-pot cyclocondensation reaction using benzaldehyde, ethyl acetoacetate, and urea in acetonitrile under reflux with lithium bromide (LiBr) as a catalyst. The reaction proceeded for 4 h, after which the solid product was filtered, washed, and recrystallized to yield DHPM with an overall yield of 67%, which was then identified and confirmed by comparison with authentic samples (De et al. 2010, Kalita and Phukan 2007).

For formulation, DHPM (2 mg/mL) was dispersed in a 10% aqueous solution of polysorbate 20 containing 5% ethanol using a homogenizer. The mixtures were then stirred for 15 min at room temperature. Radiolabeling of DHPM with [^99m^Tc]TcO_4_^−^ was achieved using stannous chloride as a reducing agent under controlled conditions with an efficiency of over 95% at room temperature. The resulting ^99m^Tc–DHPM was evaluated for particle size, in vitro stability, and in vivo lung retention in rats and rabbits (De et al. 2010).

#### Chemical aspects of [^99m^Tc]Tc-DHPM

The [^99m^Tc]TcO₄⁻ in a 2-butanone solution was evaporated under a nitrogen stream to eliminate any remaining solvent. The dried residue was rinsed with nitrogen-purged water. Subsequently, a stannous chloride solution was added at neutral pH, followed by the addition of the DHPM solution; the pH was adjusted to 7.8–8.2 for 5 min and then maintained at 7.0–8.0 under a nitrogen atmosphere (De et al. 2010).

The labeling efficiency and radiochemical purity of the final complex were confirmed using thin-layer chromatography. No detectable decomposition of the [^99m^Tc]Tc-DHPM complex was observed within 24 h at room temperature (De et al. 2010).

#### *In* vitro evaluation

The DHPM formulation was stored refrigerated in a polysorbate 20 solution. Particle sizes of the formulated preparation were measured in five different preparations, revealing a narrow size distribution with approximately 85% of particles ranging between 10 and 70 μm, and an average size of 40 μm (De et al. 2010).

Plasma protein binding of the radiopharmaceutical was evaluated in vitro via protein precipitation using 10% trichloroacetic acid (TCA) in heparinized blood. The results demonstrated a protein binding rate of 33% in rat serum (De et al. 2010).

The compound exhibited a partition coefficient (log P) of 1.45 at physiological pH (7.4), with negligible variation across the pH range of 7.0–7.4. Previous studies have suggested that lipophilic cationic complexes may be favorable for lung scintigraphy (Tsopelas et al. 2003, Palecanda and Kobzik 2001). Consistent with this suggestion, [^99m^Tc]Tc-DHPM (log *P* = 1.45 at pH 7.4) was evaluated as a candidate radiotracer for lung imaging (De et al. 2010).

HPLC analyses confirmed the radiochemical stability of [^99m^Tc]Tc-DHPM, with more than 95% of the complex remaining intact at room temperature for at least 24 h after radiolabeling (De et al. 2010).

#### In vivo evaluation

Ten female Sprague-Dawley rats (250–300 g) received an intravenous dose of 500 µCi of [^99m^Tc]Tc-DHPM complex through a 0.5 mm polyethylene (PE) catheter inserted into the femoral vein while under anesthesia. Animals were sacrificed at predefined time points (2, 5, 15, 30, 60, and 120 min) in accordance with approved ethical protocols (Table 1 of the original study). Blood and urine samples were collected by cardiac puncture and bladder aspiration, respectively. Clearance curves were generated based on blood radioactivity data, which demonstrated approximately 0.5% of the injected dose per milliliter of blood at 2 min post-injection, decreasing to 0.02% at 120 min. Blood retention was low, with radioactivity decreasing from ~ 0.5%ID/mL at 2 min to ~ 0.02% at 120 min, consistent with progressive renal excretion (De et al. 2010).

Imaging studies were conducted on mature white female rabbits (2.5–3.0 kg) using a gamma camera (GE Hawkeye). Each rabbit received 5 mCi of [^99m^Tc]Tc-DHPM in 0.5 mL via the femoral vein, and planar images were acquired at scheduled time points for up to 1 h post-injection.

The particle size distribution of [^99m^Tc]Tc-DHPM (10–70 μm) was comparable to that of macroaggregated albumin (MAA), supporting its potential for effective lung accumulation. In rats, lung uptake was high (reported in the original study as %ID/g; e.g., ~ 10.1%ID/g at 2 min and ~ 5.0%ID/g at 60 min), with lung-to-liver ratios decreasing over time but remaining > 1 during the first hour. Biodistribution analysis revealed predominant accumulation in the lungs, with minimal uptake in the liver and heart. Notably, high lung uptake remained for up to 1 h before a gradual decline, consistent with the patterns seen in other clinically used lung imaging radiopharmaceuticals (De et al. 2010).

In summary, these findings support the potential of [^99m^Tc]Tc-DHPM as a candidate for lung perfusion imaging. However, further investigations on DHPM derivatives are required to establish dose standardization (including particle number specifications), reproducibility, and performance in disease-relevant models prior to clinical translation.

#### Critical appraisal and translational relevance

Strength: Particle size distribution is within a lung-trapping range (≈ 85% between 10 and 70 μm; mean ~ 40 μm), and the complex shows high radiochemical purity/stability (≥ 95% by TLC/HPLC; stable up to 24 h).

Key in vivo signal: Rat biodistribution demonstrates strong early lung uptake and retention during the first hour, supported by rabbit scintigraphy.

Standardization gap: Particle number per administered dose and batch-to-batch reproducibility specifications are not reported, which limits direct comparison to MAA clinical benchmarks.

Workflow consideration: Although labeling is feasible at room temperature, the formulation and preparation process involves solvent handling, nitrogen-purged steps, and pH control, which may complicate routine kit-based clinical implementation.

Evidence gap: Data remain preclinical (rats/rabbits) with no human perfusion studies and no demonstrated performance in pulmonary embolism models (De et al. 2010).

Overall, ^99m^Tc–DHPM shows high preclinical promise among non-blood-derived candidates, but requires dose standardization (including particle number specifications) and clinical/disease-model validation prior to translation.

### [^99m^Tc]Tc–starch-based radiopharmaceutical

Franck Lacoeuille et al. developed a starch-based technetium-99m radiopharmaceutical for lung perfusion scintigraphy as an alternative to macroaggregated albumin (MAA). Native potato starch is a biodegradable, water-insoluble material composed of relatively uniform microparticles ranging from 10 to 100 μm in diameter. As native starch exhibits poor binding affinity for ^99m^Tc, the researchers modified the surface of the starch microparticles by oxidizing the starch and coupling it with natural polyamines. The resulting suspensions were formulated into ready-to-use kits, allowing for convenient one-step labeling procedures. This approach yielded a non-albumin, kit-formulated microparticulate tracer with preclinical characteristics compatible with lung perfusion imaging, warranting further translational evaluation (Lacoeuille et al. 2010).

#### Chemical aspects of [99mTc]Tc‑starch

After size fractionation by sieving, starch-based microparticles were oxidized with sodium periodate to yield dialdehyde starch, and cadaverine (a natural polyamine) was then covalently attached to introduce chelating functionalities suitable for ^99m^Tc labeling (Lacoeuille et al. 2010).

Ready-to-use kits were formulated under an oxygen-free (argon) atmosphere by combining 20 mg of starch-based microparticles, 50 µg of SnCl_2_, and 1 mL of 0.9% NaCl in a sterile vial. The mixture was then freeze-dried. For radiolabeling, 4 mL of sodium pertechnetate solution (200 MBq to 10 GBq) was added directly to the lyophilized kit to obtain the labeled microparticle suspension (Lacoeuille et al. 2010).

#### In vitro evaluation

Particle size distribution was evaluated using a Multisizer counting analyzer in conjunction with software-aided micrograph analysis. The results displayed a size range of 7–63 μm, with approximately 80% of particles falling within the 10–30 μm range. Using the counting analyzer, the distribution extended to ~ 5–74 μm and estimated a higher fraction of particles < 10 μm than micrograph-based measurements (Lacoeuille et al. 2010).

The radiochemical purity of the starch-based microparticles consistently exceeded 95% as assessed by 5-µm filtration, with different amounts of added pertechnetate. Labeling stability was maintained for at least 8 h under standard storage conditions (25 °C), with only 2–3% loss of RCP after 24 h. Furthermore, the formulation stability seemed satisfactory even under more challenging conditions, showing negligible loss of radiochemical purity after prolonged incubation with histidine or human plasma at 37 °C (4% loss after 3 h with histidine; 1% loss after 1 h in human plasma) (Lacoeuille et al. 2010).

#### In vivo evaluation

In scintigraphy studies, 15 MBq of labeled starch-based microparticles (*n* = 3) or [⁹⁹ᵐTc]Tc-MAA (control, *n* = 3) were injected into anesthetized healthy Wistar rats, followed by dynamic and static planar acquisitions to generate the time–activity curve (Lacoeuille et al. 2010).

For biodistribution, rats were sacrificed at 15 min (*n* = 4) or 2 h (*n* = 4) after intravenous injection, and organs were excised, washed, weighed, and counted; lung activity exceeded 80% of the injected dose at 15 min, while liver activity remained 2.4%ID (Table 1 of the original study) (Lacoeuille et al. 2010).

In metabolism studies, rats injected with starch-based microparticles (*n* = 3) or sodium pertechnetate (control, *n* = 1) were housed in individual metabolism cages for 12 h to assess urinary excretion and radiolabel fate by gel filtration chromatography (Lacoeuille et al. 2010).

The radiotracer showed consistent pulmonary uptake, allowing lung imaging to be performed for up to 90 min. Clearance occurred mainly through urinary excretion, supported by radioactivity accumulation in the bladder and kidneys, with minimal liver activity (~ 2.4%) (Lacoeuille et al. 2010).

Dynamic acquisition showed a shorter lung biological half-life for the starch tracer than for MAA (3.2 ± 0.5 h vs. 13.1 ± 8.6 h, respectively) (Lacoeuille et al. 2010). The lung-to-vascular, lung-to-liver, and lung-to-stomach activity ratios remained stable at both 15 and 30 min post-injection, with more than 80% of the injected dose localized in the lungs at 15 min (lung/vascular ≈ 900–1,100; lung/liver ≈ 90; lung/stomach ≈ 150–180 at 15–30 min) (Lacoeuille et al. 2010).

This starch-based radiotracer offers several practical advantages over conventional particulate agents for lung perfusion imaging. Its ready-to-use, lyophilized kit enables single-step labeling, facilitating a kit-based radiopharmacy workflow. The formulation demonstrated high radiochemical purity, good in vitro stability, and favorable preclinical biodistribution with high early lung uptake and low hepatic activity. Future studies are warranted to validate efficacy and safety in humans; prior to first-in-human studies, the authors highlight the need for evaluation in pulmonary embolism animal models and acute toxicity assessments.

#### Critical appraisal and translational relevance

Strength: Ready-to-use, lyophilized kit enables one-step labeling with high radiochemical purity and stability; particle size distribution is largely within a lung-trapping range (e.g., ~ 80% of particles in the 10–30 μm fraction by micrograph analysis), supporting practical radiopharmacy feasibility.

Key in vivo signal: In rats, > 80% of the injected dose localized in the lungs at 15 min with very low liver activity (~ 2.4%ID), high and stable lung-to-background ratios, and lung imaging feasible up to ~ 90 min.

Standardization gap: Particle number per administered dose and batch-to-batch clinical specifications are not reported; the small-particle fraction (< 10 μm) varies depending on the sizing method (counting analyzer vs. micrograph), which may affect reproducibility and extrapulmonary distribution.

Evidence gap: Evidence remains preclinical (healthy rats) with no human perfusion studies; the authors note the need for pulmonary embolism model evaluation and acute toxicity assessment before phase I translation (Lacoeuille et al. 2010).

Overall, ^99m^Tc starch-based microparticles show high translational promise among non-blood-derived candidates due to pharmacopeia-compatible lung uptake and kit-based preparation, but require dose standardization (including particle number specifications) and disease-model/toxicity validation prior to clinical translation.

### [^99m^Tc]Tc-zolmitriptan

H. M. Rashed and colleagues developed and evaluated a novel technetium-99m (^99m^Tc)-labeled zolmitriptan formulation for lung scintigraphy. Zolmitriptan (a selective serotonin receptor agonist) was radiolabeled with ^99m^Tc via a direct labeling approach under reductive conditions, motivated by prior observations that lungs can act as a reservoir for compounds with affinity to serotonergic targets/transporters. The study investigated different parameters influencing labeling efficiency, ultimately achieving an optimal radiolabeling yield of 92.5 ± 0.61%. The optimized conditions corresponded to 2 mg zolmitriptan, 30 mg Na_2_S_2_O_4_, pH 6, 25 ± 2 °C, and 45 min reaction time (Rashed et al. 2016).

#### Chemical aspects of [99mTc]Tc‑zolmitriptan

In a 10 mL vial, 600 µL of zolmitriptan solution (0.5–3 mg in distilled water) was mixed with 500 µL of freshly prepared Na_2_S_2_O_4_ solution (2–50 mg in water). Then, 20 µL of ^99m^TcO_4_− (51.5 MBq) was added, the pH was adjusted using 0.05 M NaHCO_3_, and the mixture was shaken and incubated under varying temperatures and reaction times (5–120 min). Radiochemical species were quantified as ^99m^Tc-zolmitriptan, free ^99m^TcO_4_−, and reduced hydrolyzed ^99m^TcO_2_ colloid using paper chromatography/TLC and paper electrophoresis (Rashed et al. 2016).

#### In vitro evaluation

The radiochemical yield and in vitro stability of ^99m^Tc-zolmitriptan were assessed using ascending paper chromatography/TLC with two mobile-phase systems, with paper electrophoresis used as a confirmatory method to support species assignment. The maximum labeling yield of 92.5% ± 0.61% was achieved after a 45-minute reaction at 25 ± 2 °C. The complex remained stable for up to 24 h in vitro. Optimal radiochemical yield was obtained at pH 6, while lower yields were observed under highly acidic (pH 3) or alkaline (pH 10) conditions (pH 3 ≈ 53.33%, pH 10 ≈ 50.33%), likely due to competing redox and hydrolytic side reactions (Rashed et al. 2016).

The authors note that using Na_2_S_2_O_4_ avoids stannic-oxide–related colloidal interference associated with stannous-chloride-based labeling, with ^99m^TcO_2_ being the main reduced/hydrolyzed species considered in QC (Rashed et al. 2016).

#### In vivo evaluation

Biodistribution was evaluated in male Swiss albino mice (20–25 g), with %ID/g (mean ± SD) reported at 0.25, 0.5, 1, 2, and 4 h post-injection (*n* = 3 per time point) following intravenous administration of 150 µL containing 6.2 MBq (Rashed et al. 2016).

[^99m^Tc]Tc-zolmitriptan showed rapid pulmonary uptake, with lung activity peaking at 30 min (reported in the original study as %ID/g: 23.89 ± 1.2%ID/g) and remaining relatively high at 1 h (17.305 ± 1.6%ID/g), while blood activity declined over time, supporting an imaging window within the first hour. Renal uptake was prominent (kidney 24.85 ± 1.8%ID/g at 30 min), consistent with predominant urinary excretion. Aside from renal/urinary activity and moderate background uptake (e.g., liver ~ 6–7%ID/g around 30–60 min), most other tissues showed lower activity than lungs during the first hour, consistent with favorable lung contrast (Rashed et al. 2016).

Gamma scintigraphy was performed using a Siemens dual-head gamma camera with a 5-mm pinhole collimator at 140 keV (20% window), acquiring 10-min static images (512 × 512 matrix) at 0.25–4 h post-injection (Rashed et al. 2016).

As a non-blood-derived, non-particulate compound, ^99m^Tc-zolmitriptan may mitigate concerns associated with human-serum–derived products such as ^99m^Tc-MAA, while providing substantial lung uptake in mice (Rashed et al. 2016).

Stomach activity was not dominant relative to lung uptake at early time points, which the authors interpreted as consistent with acceptable in vivo stability. Nevertheless, direct in vivo assessment of the free pertechnetate fraction would strengthen this conclusion. The authors also note that the observed lung uptake is higher than values they cite for several previously explored agents (e.g., 123I/125I-IPMPD, ^99m^Tc(CO)_5_I, and ^99m^Tc-DHPM), although cross-study comparisons should be interpreted cautiously (Rashed et al. 2016).

Overall, these results support further evaluation of ^99m^Tc-zolmitriptan, including disease-relevant models, dosimetry/toxicity assessment, and head-to-head comparison versus ^99m^Tc-MAA under matched conditions (Rashed et al. 2016).

#### Critical appraisal and translational relevance

Strength: Non-blood-derived, non-particulate, small-molecule tracer with optimized radiolabeling yield (92.5 ± 0.61%) and in vitro stability reported up to 24 h; QC explicitly differentiates complex, free pertechnetate, and reduced/hydrolyzed species.

Key in vivo signal: In mice, lung uptake peaked at 30 min (23.89 ± 1.2%ID/g) and remained relatively high at 1 h (17.305 ± 1.6%ID/g), with scintigraphy confirming prominent lung visualization during the early imaging window.

Standardization gap: As a non-particulate agent, particle number/size specifications do not apply; however, translational standardization is still incomplete (e.g., kit-style reproducibility, robustness of QC across batches, and quantitative lung-to-background benchmarks versus MAA under matched conditions).

Evidence gap: Evidence remains preclinical (normal mice) with no pulmonary embolism disease model and no human perfusion data; dosimetry/toxicity assessment and head-to-head comparison with ^99m^Tc-MAA would be needed before translation (Rashed et al. 2016).

Overall, ^99m^Tc-zolmitriptan shows strong preclinical lung uptake and imageability among non-blood-derived candidates, but requires disease-model validation, standardized preparation/QC, and clinical safety/efficacy evaluation before it can be considered a realistic alternative to ^99m^Tc-MAA.

In the following section, we discuss lung-relevant chitosan-based platforms that have been explored in the context of pulmonary scintigraphy; however, their performance as true intravenous perfusion tracers has not yet been established.

**Fig. 2:**
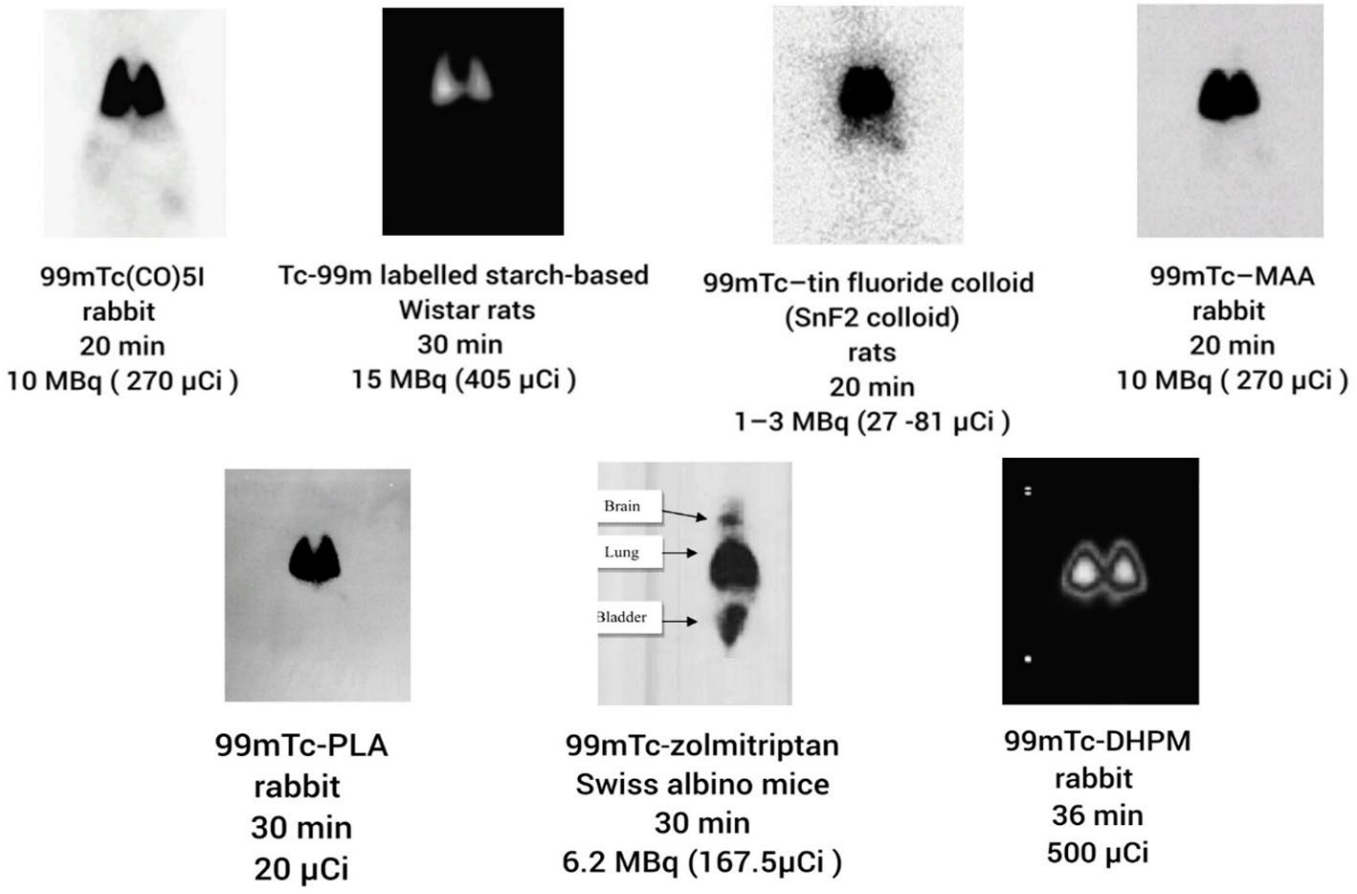
Representative planar scintigraphic images illustrating typical biodistribution patterns of selected ^99m^Tc-labeled tracers in animal models. **A** rabbit, [^99m^Tc]Tc(CO)_5_I, 20 min (10 MBq). **B** Wistar rats, [^99m^Tc]Tc-starch-based microparticles, 30 min (15 MBq). **C** rats, [^99m^Tc]Tc–SnF_2_ colloid, 20 min (1–3 MBq). **D** rabbit, [^99m^Tc]Tc-MAA, 20 min (10 MBq). **E** rabbit, [^99m^Tc]Tc-PLA microspheres, 30 min (20 µCi). **F** Swiss albino mice, [^99m^Tc]Tc-zolmitriptan, 30 min (6.2 MBq). **G** rabbit, [^99m^Tc]Tc-DHPM, 36 min (500 µCi). Panels are adapted from the original publications

Table 1 summarizes key quality attributes, workflow practicality, and translational barriers across the reviewed ^99m^Tc candidates, while Fig. 2 provides representative planar scintigraphic images illustrating typical lung-dominant and extrapulmonary uptake patterns. Further data are available in the Supplementary Tables.

**Table 1:** Translational readiness matrix for non-blood-derived ^99m^Tc lung perfusion candidates versus [^99m^Tc]Tc-MAA

Tracer	Clinical status	Perfusion-relevant mechanism	Specs/QC suitable for translation (dose unit, acceptance criteria)	Kit-like workflow (time-to-dose, practicality)	Validation level (disease model/humans)	Overall readiness (qualitative)	Key limitation/driver for alternatives
[^99m^Tc]Tc-MAA (25)	Clinical standard	✓	✓	✓	✓	**N/A (clinical standard); human-derived source motivates alternatives**	Human-derived + particle-related safety/variability (motivates alternatives)
[^99m^Tc]Tc-PLA microspheres (13)	Preclinical	✓ (trapping plausible)	✗ (particle number NR; broad PSD)	✗ (~ 120 min, pH steps)	✗ (no PE model; no humans)	**Low**	Not standardized (dose/PSD) + workflow not practical
[^99m^Tc]Tc-SnF₂ colloid (lung formulation) (40)	Preclinical for lung	✓ (particle-based trapping)	△ (RCP high; dose particle specs NR; reproducibility unclear)	△ (~ 50 min mixing)	✗ (no PE model; no humans)	**Low-Moderate**	Needs dose specs + batch reproducibility + clinical validation
[^99m^Tc]Tc(CO)₅I (16)	Preclinical	△ (mechanism not fully established)	△ (RCP by HPLC; but clinical-type specs lacking)	✗ (autoclave/high P/T)	✗ (no PE model; no humans)	**Low**	Manufacturing impractical + lung retention below MAA benchmark
[^99mTc^]Tc-DHPM (microparticulate formulation) (17)	Preclinical	✓ (particle-based trapping likely)	△ (PSD reported; particle number/dose NR)	△ (solvent/N₂ handling; formulation complexity)	✗ (no PE model; no humans)	**Low-Moderate**	Missing dose standardization + limited translational QC/validation
[^99mTc^]Tc-starch microparticles (kit) (19)	Preclinical	✓	△ (RCP good; PSD method-dependent; particle number/dose NR)	✓ (lyophilized kit; one-step label)	✗ (no PE model; no humans)	**Moderate**	Closest workflow-wise, but needs dose specs + disease/toxicity + humans
[^99m^Tc]Tc-zolmitriptan (20)	Preclinical	△ (non-particulate; lung uptake ≠ perfusion proof)	△ (labeling/QC reported; kit robustness NR)	△ (~ 45 min; reagent freshness/pH control)	✗ (no PE model; no humans)	**Low-Moderate**	Needs mechanistic + disease-model + head-to-head vs. MAA + clinical safety

Interpretation note: High pulmonary uptake alone is insufficient for clinical translation. Across the reviewed candidates, the most common barriers were the absence of standardized dose units (e.g., particle number), incomplete particle-size specifications and acceptance criteria, lack of kit-compatible workflows with routine QC, and limited validation in disease-relevant models (e.g., pulmonary embolism) and humans. Consequently, translational readiness remains low to moderate for most non-blood-derived ^99m^Tc agents despite promising preclinical signals

QC, quality control; RCP, radiochemical purity; RPSD, radioactive particle size distribution; NR, not reported; %ID, percentage of injected dose; %ID/g, percentage of injected dose per gram; PE, pulmonary embolism; PH, pulmonary hypertension; RES, reticuloendothelial system

✓ = meets key expectations; △ = partially/insufficiently reported; ✗ = not met or not reported; N/A = not applicable

Biodistribution metrics are reported as in the original studies (e.g., %ID/g for DHPM and zolmitriptan; %ID/organ where available)

### Chitosan-based platforms (lung-relevant only)

Chitosan is a cationic biopolymer obtained by deacetylation of chitin and is composed of β-(1→4)-linked glucosamine units, providing primary amine groups that enable extensive chemical functionalization (Sorlier et al. 2001, Austin 1988, Lubben et al. 2001, Younes and Rinaudo 2015). From a translational standpoint, a key limitation is the lack of full pharmaceutical standardization across suppliers with respect to molecular weight and degree of deacetylation, which can drive batch-to-batch variability in formulation behavior and in vivo performance (Lubben et al. 2001, Zadeh Mehrizi et al. 2023).

For lung perfusion scintigraphy, chitosan should be viewed as a platform rather than a single tracer. To be clinically competitive with [^99m^Tc]Tc-MAA, any chitosan-based candidate would need (i) a clearly defined mechanism that reflects regional perfusion (e.g., controlled mechanical trapping in the pulmonary microvasculature or a validated alternative non-occlusive binding mechanism), (ii) tight specifications for size distribution and administered dose units (particle number or equivalent), and (iii) reproducible kit-style preparation with practical QC—requirements that are frequently absent or inconsistently reported for experimental nano/microparticle systems (Lubben et al. 2001, Zadeh Mehrizi et al. 2023).

Chitosan-based nanoparticles have been widely investigated as drug-delivery carriers because their size, surface charge, and functional groups can be tuned to modulate biodistribution, cellular interactions, and residence time, and they can also be radiolabeled with ^99m^Tc for scintigraphic tracking of formulation fate (Agnihotri et al. 2004, Nagpal et al. 2010, Ali and Ahmed 2018, Franca et al. 2020, Elkomy et al. 2022, Banerjee et al. 2002). However, most nano-chitosan systems were developed for targeted delivery (e.g., antimicrobial/oncologic applications) rather than IV perfusion mapping, so lung retention or tissue uptake in these studies should not be interpreted as evidence of perfusion-tracer equivalence without dedicated validation in a perfusion-relevant disease model (Agnihotri et al. 2004, Nagpal et al. 2010, Banerjee et al. 2002).

A lung-relevant nano-direction has been explored via cationic radiolabeled nanomaterials designed to accumulate rapidly in the pulmonary capillary bed through electrostatic interactions with negatively charged endothelial glycocalyx components rather than via capillary microembolization (Lobov et al. 2013, Reitsma et al. 2007). This concept is attractive because it aims to achieve prominent lung localization while avoiding deliberate microvascular obstruction. However, it also shifts the burden of proof toward demonstrating that the signal truly maps perfusion (and not merely charge-mediated endothelial binding), particularly under disease conditions such as pulmonary embolism (Lobov et al. 2013, Reitsma et al. 2007).

Separately, chitosan has been widely used as a surface modifier for inhaled particulate systems (Rasul et al. 2020). For example, chitosan-coated PLGA nanoparticles (evaluated in the context of pulmonary antifungal delivery) were reported to show improved lung retention and favorable gamma-scintigraphic visualization compared with uncoated systems, consistent with enhanced mucosal interaction and altered deposition/clearance behavior (Paul et al. 2018). While such data support the feasibility of radiolabeling and lung retention for chitosan-coated carriers, these inhalation-oriented studies do not automatically establish suitability as IV perfusion tracers, and their clinical relevance for perfusion defect detection remains to be demonstrated (Paul et al. 2018).

Overall, the main reasons chitosan-based systems have not progressed toward routine clinical lung perfusion imaging mirror broader translational barriers observed across experimental tracers: incomplete material standardization (MW/DD), insufficient dose specifications (particle number or equivalent), limited batch-to-batch reproducibility and QC frameworks, and a lack of validated performance in disease-relevant models and head-to-head comparisons against [^99m^Tc]Tc-MAA (Lubben et al. 2001, Zadeh Mehrizi et al. 2023, Lobov et al. 2013, Paul et al. 2018).

#### Critical appraisal and translational relevance

Strength: Chitosan is a versatile, modifiable platform with established radiolabeling feasibility and tunable surface properties; lung-localizing cationic nanomaterial concepts suggest a potential non-occlusive direction for future research (Lubben et al. 2001, Lobov et al. 2013).

Key in vivo signal: Selected nano/cationic systems have demonstrated rapid pulmonary localization, and chitosan-coated inhaled systems have shown prolonged lung retention in scintigraphic evaluation (Lobov et al. 2013, Paul et al. 2018).

Standardization gap: Pharmaceutical standardization remains challenging (MW/DD variability), and clinically meaningful specifications (dose units, particle number, reproducibility, and routine QC acceptance criteria) are often not established (Lubben et al. 2001, Zadeh Mehrizi et al. 2023).

Evidence gap: The current evidence is preclinical and heterogeneous, with limited disease-model validation (e.g., PE) and no human perfusion studies; mechanistic distinction between perfusion mapping and non-specific endothelial/mucosal retention requires rigorous validation (Lobov et al. 2013, Paul et al. 2018).

Overall, chitosan-based systems are best positioned as future platform candidates rather than near-term alternatives to [^99m^Tc]Tc-MAA for routine lung perfusion scintigraphy, pending standardization and disease-model/clinical validation (Lubben et al. 2001, Zadeh Mehrizi et al. 2023, Lobov et al. 2013, Paul et al. 2018).

## Discussion

This review shows that non-blood-derived ^99m^Tc lung perfusion tracers have advanced substantially at the preclinical level; however, their translation into routine clinical practice remains limited. Across the reviewed candidates, the central challenge is not merely achieving pulmonary uptake, but achieving uptake through a mechanism that is physiologically interpretable for perfusion imaging, operationally practical in routine radiopharmacy, and compatible with regulatory-quality expectations (Hunt et al. 2006, Perkins and Frier 1999).

A major distinction emerging from the reviewed literature is the contrast between particle-based and non-particulate approaches. Particle-based tracers (e.g., PLA microspheres, lung-formulated [^99m^Tc]Tc–SnF_2_ colloid, DHPM microparticulate formulations, and starch-based microparticles) are conceptually closer to [^99m^Tc]Tc-MAA because they rely on pulmonary microvascular trapping. This mechanism is already clinically established for perfusion scintigraphy and provides a direct physiological basis for regional perfusion mapping. Nevertheless, most particulate alternatives remain underdeveloped from a translational standpoint, particularly with respect to standardized particle number per administered dose, acceptance criteria for particle-size distribution, and batch-to-batch reproducibility (Ergun et al. 2000, De et al. 2010).

By contrast, non-particulate/small-molecule tracers (e.g., [^99m^Tc]Tc(CO)_5_I and ^99m^Tc-zolmitriptan) demonstrate that prominent lung localization can be achieved without particle trapping. These agents are scientifically important because they may enable non-occlusive strategies and potentially avoid some particle-related limitations. However, pulmonary uptake alone does not establish perfusion specificity. For clinical translation, such tracers must demonstrate that their signal reflects regional pulmonary perfusion rather than alternative determinants such as receptor affinity, endothelial interaction, or lipophilic tissue partitioning. This mechanistic distinction becomes especially important in disease-relevant settings such as pulmonary embolism, where tracer behavior must remain interpretable under altered pulmonary hemodynamics (Hunt et al. 2006, Miroslavov et al. 2009).

Another recurrent theme is the gap between preclinical biodistribution performance and radiopharmacy practicality. Several candidates show encouraging lung uptake but depend on workflows that are difficult to implement in routine nuclear medicine settings, including prolonged mixing/incubation, low-pH multistep procedures, nitrogen-purged preparation, or high-pressure/high-temperature synthesis. In contrast, the enduring clinical success of [^99m^Tc]Tc-MAA reflects not only effective first-pass lung trapping but also a well-established kit-based preparation process, straightforward quality control, and operational compatibility with routine practice. Within this context, starch-based microparticles appear particularly promising because they combine high early lung uptake with a lyophilized, one-step labeling format that is more aligned with real-world radiopharmacy workflows (Lacoeuille et al. 2010, Dewanjee 1990).

The findings of this review also support a more balanced framing of the clinical motivation for alternatives to [^99m^Tc]Tc-MAA. Historical concerns related to blood-derived products have contributed to interest in albumin-free tracers; however, contemporary manufacturing controls and regulatory oversight have substantially reduced the practical risk of pathogen transmission. Accordingly, the rationale for alternative tracers should not be framed solely in terms of biological-source safety. Additional drivers include improved standardization, reproducible dose specifications, supply-chain resilience, platform flexibility, and the opportunity to develop agents with more predictable pharmacokinetics or simpler preparation workflows (Hunt et al. 2006, Miniati et al. 1996, Delgado et al. 2000, Whinnery and Young 1980).

From a translational and regulatory perspective, the most consistent barrier across candidates is the absence of a pharmacopeia-aligned development framework. For particulate tracers, this includes standardized dose units (especially particle number per dose), validated particle-size acceptance ranges, radiochemical purity criteria, and routine batch quality-control procedures. For non-particulate tracers, priorities include robust mechanistic validation, standardized quantitative imaging benchmarks, and reproducible preparation methods suitable for clinical radiopharmacies. In both categories, head-to-head comparisons with [^99m^Tc]Tc-MAA under matched experimental conditions are essential to generate clinically interpretable evidence (Canziani et al. 2022, European ).

Based on the current evidence, a staged roadmap for future development can be proposed. First, candidate tracers should be evaluated in standardized healthy-animal protocols using harmonized endpoints (e.g., lung uptake, liver/spleen uptake, lung-to-background ratios, and retention kinetics). Second, the most promising tracers should be tested in pulmonary embolism or other perfusion-defect models to establish disease relevance. Third, formulation and QC specifications should be defined early, including reproducibility, acceptance criteria, and kit compatibility. Fourth, acute toxicity, dosimetry, and safety studies should be completed before early-phase human translation. Such a stepwise framework would improve cross-study comparability and facilitate identification of candidates with realistic clinical potential (Ergun et al. 2000, Perkins and Frier 1999).

Overall, no currently reported non-blood-derived tracer is ready to replace [^99m^Tc]Tc-MAA in routine clinical lung perfusion imaging. Nonetheless, several platforms—particularly kit-formulated biodegradable microparticles—show meaningful translational potential. Future progress will depend less on demonstrating lung uptake alone and more on integrating mechanism, standardization, workflow practicality, and clinical validation into a coherent translational strategy.

## Conclusion

In this review, we synthesized the available evidence on non-blood-derived ⁹⁹ᵐTc lung perfusion candidates and mapped each agent against practical translational benchmarks (mechanism, dose specification/QC, workflow feasibility, and validation level).

Overall, the reviewed non-blood-derived ^99m^Tc candidates demonstrate that substantial pulmonary localization can be achieved in preclinical models using both particulate and non-particulate strategies. Nevertheless, translation beyond proof-of-concept remains limited because most agents lack at least one key requirement relative to [^99m^Tc]Tc-MAA: standardized dose units (particle number/size distribution), robust routine QC criteria, kit-compatible workflow, and validated performance in disease-relevant models and/or humans.

Among the particulate approaches, kit-formulated biodegradable microparticles (e.g., starch-based systems) appear comparatively closer to clinical practicality, whereas small-molecule approaches (e.g., [^99m^Tc]Tc-zolmitriptan or [^99m^Tc]Tc(CO)_5_I) require stronger mechanistic validation and head-to-head benchmarking. Chitosan-based systems are best positioned as modifiable platforms for future innovation rather than near-term replacements for routine IV perfusion scintigraphy. Future progress should prioritize matched comparisons versus [^99m^Tc]Tc-MAA, disease-model validation (e.g., PE), and pharmacopeia-aligned specifications to enable reproducible manufacturing and regulatory acceptance.

## Data Availability

The data supporting the conclusions of this review are derived from previously published studies and are available in the cited references. Readers are encouraged to consult the original publications for detailed methodologies and results.

## Abbreviations

⁹⁹ᵐTc: Technetium-99 m
PE: Pulmonary Embolism
MAA: Macroaggregated Albumin
QC: Quality Control
Biodistribution: Distribution of a radiopharmaceutical within tissues/organs
IV: Intravenous
SPECT: Single Photon Emission Computed Tomography
CT: Computed Tomography
iTLC: Instant thin-layer chromatography
